# Mapping of Patellar Fracture Patterns: A Multicenter Study of 237 Patients

**DOI:** 10.3390/jcm14041335

**Published:** 2025-02-17

**Authors:** Julia Elisabeth Lenz, Amadeus Dominik Schraag, Luis Plank, Christian von Rüden, Volker Alt, Johannes Weber

**Affiliations:** 1 Department of Trauma Surgery, University Hospital Regensburg, Franz-Josef-Strauß-Allee 11, 93053 Regensburg, Germany; 2 Department of Trauma Surgery, Orthopaedics and Hand Surgery, Klinikum Weiden, Söllnerstraße 16, 92637 Weiden, Germany; 3 Institute for Biomechanics, Paracelsus Medical University, Strubergasse 21, 5020 Salzburg, Austria

**Keywords:** patella, fracture, mapping, computed tomography (CT), AO/OTA classification

## Abstract

**Background/Objectives:** Patellar fractures are rare but clinically significant due to their impact on knee function. These injuries vary from simple transverse to complex comminuted patterns. Computed tomography (CT) offers superior visualization compared to radiographs, enabling accurate classification and surgical planning. This study utilized CT-based fracture mapping to analyze fracture patterns and evaluate the impact of age, trauma-center level, and AO/OTA classification. **Methods:** This retrospective study included 237 patients diagnosed with patellar fractures who underwent CT imaging. Fractures were classified using the AO/OTA system, and fracture mapping was performed by overlaying fracture lines onto a standardized template. Statistical analysis assessed correlations between patient demographics, trauma-center level, and fracture patterns. **Results:** The cohort comprised 107 males and 130 females with a mean age of 56.9 ± 20.9 years. Males were significantly younger than females (49.2 vs. 63.3 years, *p* < 0.001). Fractures were evenly distributed between the right (46%) and left (54%) patellae. Type C fractures were the most common (54.4%), followed by Type B (29.9%) and Type A (15.6%). Trauma-center level was inversely associated with fracture severity (*p* < 0.001), with complex fractures more common at lower-level centers. Age was positively correlated with fracture severity (*p* = 0.001). Fracture mapping revealed the central patella as the most frequently affected region, with transverse fractures extending medially and laterally, sparing the upper and lower poles. **Conclusions:** CT imaging enhances the classification and mapping of patellar fractures, highlighting the central patella as the primary site of injury. Fracture severity correlates with age and trauma-center level. These findings support CT-based mapping as a valuable tool for improving surgical planning and treatment outcomes.

## 1. Introduction

Patellar fractures, although constituting only approximately 1% of all skeletal injuries, have a disproportionately significant impact on knee function due to their central role in the extensor mechanism of the knee joint [1,2]. The patella serves as a key biomechanical lever that enhances the quadriceps muscle’s efficiency, facilitating activities such as walking, running, and climbing stairs. Disruption of the patella’s structural integrity can lead to severe functional impairments, including loss of extensor strength, chronic pain, and patellofemoral arthrosis, potentially resulting in substantial long-term morbidity if not adequately addressed [2,3]. These fractures most commonly occur due to direct trauma, such as falls or direct blows to the knee, often during high-impact activities or accidents. Less frequently, patellar fractures may result from indirect mechanisms, such as sudden quadriceps contraction in a flexed knee, which can generate tensile forces sufficient to cause fracture [4].

The morphology of patellar fractures can vary widely, from simple, non-displaced transverse fractures to more complex, comminuted patterns with significant displacement and joint incongruity [5]. This variability poses a challenge in both diagnosis and management, requiring a nuanced understanding of the underlying fracture mechanics and patient-specific factors. The primary goals of treatment are to restore the anatomical alignment of the patella, reestablish the extensor mechanism, and enable early mobilization to prevent joint stiffness and minimize the risk of post-traumatic osteoarthritis [6,7].

Surgical intervention, particularly open reduction and internal fixation (ORIF), remains the standard of care for displaced patellar fractures and fractures involving significant comminution [8]. Common surgical techniques include tension band wiring, modified cannulated screw cerclage, and plate osteosynthesis, with the choice of method guided by fracture type, surgeon expertise, and patient factors. Tension band wiring, for example, is often favored for simple transverse fractures, while plate osteosynthesis may be more suitable for comminuted patterns requiring robust fixation [5,6,7]. Regardless of the chosen technique, the success of these procedures is contingent on achieving stable fixation, precise anatomical reduction, and minimizing articular incongruity [1,8].

Accurate diagnosis and characterization of patellar fractures are essential for planning effective surgical interventions. Conventional diagnostic tools, such as plain radiographs, provide limited two-dimensional information, which can be insufficient for fully understanding complex fracture patterns [9,10]. This limitation is particularly relevant in multi-fragmentary fractures, where subtle variations in fracture morphology may not be readily apparent. As a result, computed tomography (CT) has gained prominence as an advanced imaging modality that offers detailed, three-dimensional visualization of fracture patterns, facilitating more accurate classification and surgical planning [11,12].

An emerging and innovative approach to fracture characterization is the use of “fracture mapping”, a technique initially developed for analyzing complex fractures in other anatomical regions, such as the tibial plateau and the distal tibia (pilon fractures) [13,14,15,16]. Fracture mapping involves tracing and overlaying fracture lines from multiple CT images to identify recurring patterns, zones of comminution, and areas of stress concentration. This method has been instrumental in elucidating biomechanical failure mechanisms, guiding surgical approaches, and improving outcomes in other fracture types. Applying this technique to patellar fractures holds great promise for advancing the understanding of their morphology and optimizing treatment strategies.

The objective of this study is to apply CT-based fracture mapping to patellar fractures to achieve a comprehensive characterization of fracture patterns. By systematically identifying consistent fracture lines, zones of comminution, and common patterns of injury, this study seeks to contribute to a deeper understanding of the biomechanical and anatomical factors underlying patellar fractures. Additionally, this research aims to explore the influence of demographic and clinical variables, such as patient age, trauma-center level, and fracture classification, on fracture morphology and treatment outcomes. By bridging the gap between advanced imaging techniques and clinical practice, this study aspires to inform the development of more effective, individualized treatment strategies that optimize functional recovery and long-term outcomes.

## 2. Materials and Methods

### 2.1. Study Population

This retrospective study included 237 patients from four hospitals, with participating centers being one Level I Trauma Center (Hospital 1), one Level II Trauma Center (Hospital 2), and two Level III Trauma Centers (Hospitals 3–4) as defined by the American College of Surgeons [17]. All patients diagnosed with a patellar fracture between 2006 and 2024 in Hospitals 2–4 as well as patients diagnosed between 2012 and 2022 in Hospital 1 who underwent a CT scan of the affected knee were considered for inclusion. Patients with inadequate quality of image were excluded.

### 2.2. Radiological Evaluation

Radiological evaluation was performed using the available CT data of the affected patella. All CT scans were analyzed using the respective radiology viewers provided by the hospitals and coronal reconstructions. The articular surface slice that best depicted the fracture pattern was selected for fracture mapping. Fractures were classified according to the AO/OTA classification system, which provides a detailed categorization of fracture types and which is still the most widely used classification system worldwide [18]. In the currently valid AO/OTA classification, fracture configurations are categorized in the axial, coronal and sagittal planes, with complex joint fracture patterns described as fully articular frontal/coronal simple (C1), wedge-shaped (C2), and multi-fragmentary (C3). However, there is no further subgroup classification for multi-fragmentary fractures describing detailed fracture patterns multi-axially.

### 2.3. Fracture Mapping

**Figure 1:**
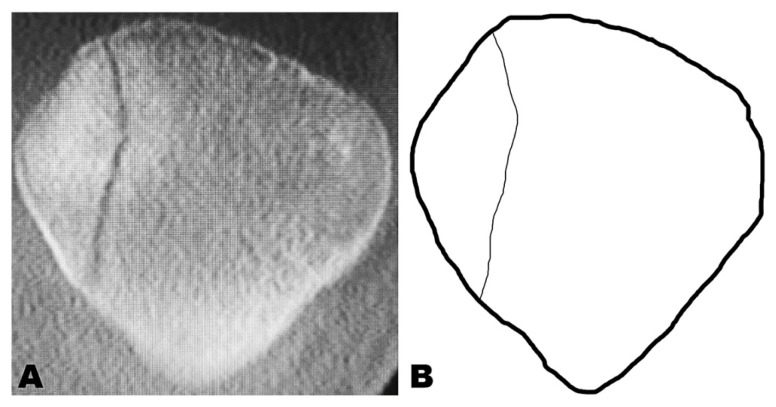
Fracture mapping of a patellar fracture. (**A**) Coronar slice of a right patella computer tomogram. (**B**) Fracture map.

Fracture mapping was performed by transferring the coronal CT slice into PowerPoint software (Microsoft Office, 2016; Microsoft Corp., Redmond, WA, USA). This method, first described by Cole et al. for pilon fractures, allows for the superimposition of multiple fracture patterns to identify consistent fracture lines and zones of comminution [13]. All fractures were scaled to a uniform size to ensure comparable representation. CT scans were mirrored if necessary to ensure that only right patellae were obtained. Fracture lines were traced and then overlaid to create a visual representation of the fracture pattern (see Figure 1).

### 2.4. Data Analysis

Statistical analysis of fracture patterns was conducted using SPSS software version 25 (SPSS Inc., Chicago, IL, USA). The frequency of different fracture patterns was determined and analyzed for their distribution. Chi-square tests were employed to assess potential correlations between fracture patterns, age, and sex, with a *p*-value of <0.05 considered statistically significant. Additionally, a linear regression model was used to examine influencing factors on the severity of the injury.

## 3. Results

**Figure 2:**
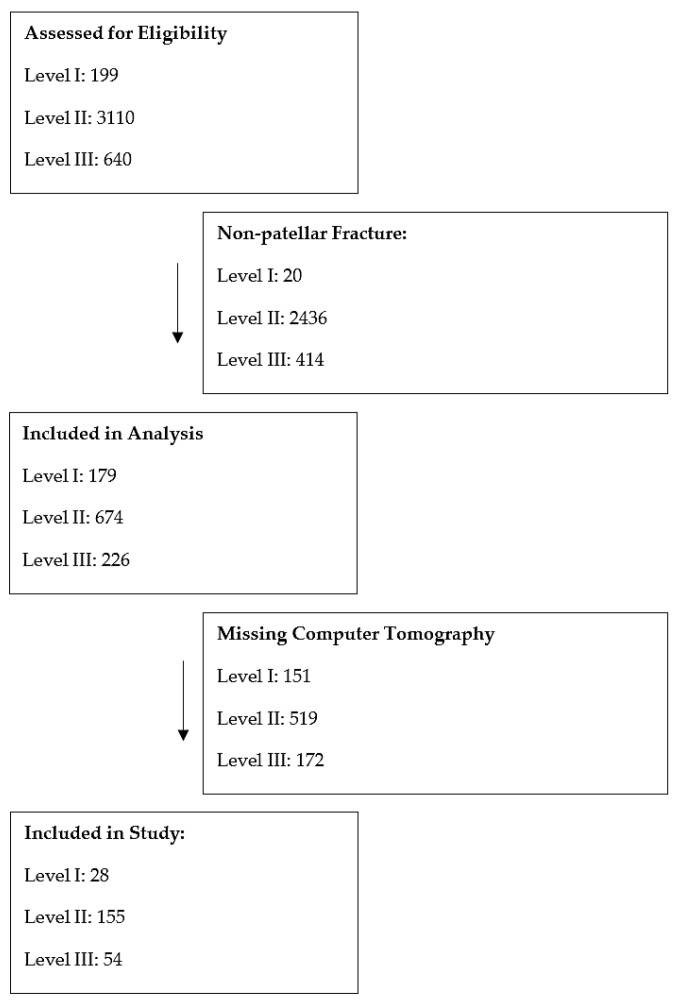
PRISMA flow diagram.

In total, 237 patellar fractures were analyzed, as demonstrated in the PRISMA flow diagram (Figure 2).

### 3.1. Demographics

**Figure 3:**
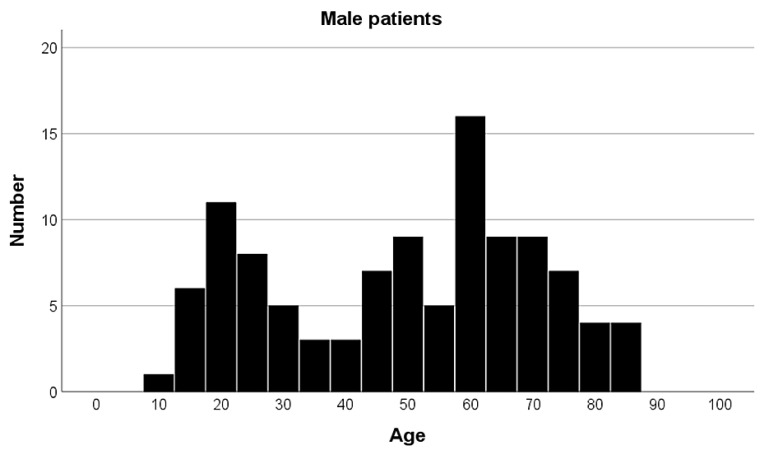
Age distribution of male patients in the cohort. Age is presented in years, number of patients as total.

**Figure 4:**
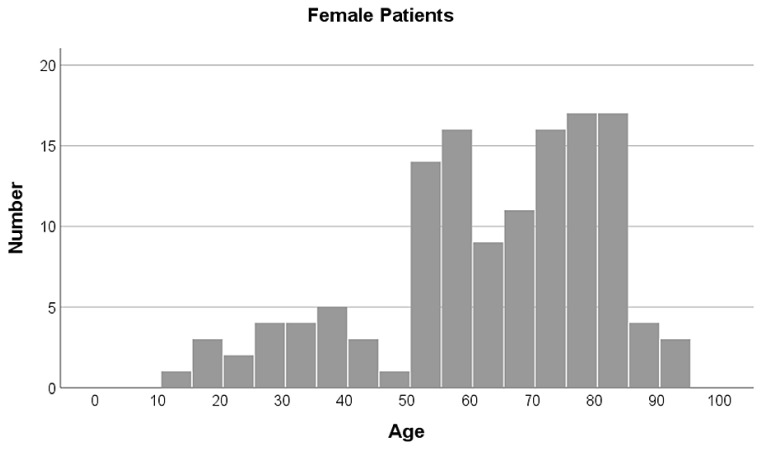
Age distribution of female patients in the cohort. Age is presented in years, number of patients as total.

The cohort consisted of 107 males and 130 females. The mean age of the patients was 56.9 ± 20.9 years (see Figure 3 and Figure 4). Male patients were significantly younger than female patients (*p* < 0.001), with the mean age for males being 49.21 ± 21.1 years and for females 63.28 ± 18.6 years. Of the cases, 46% involved the right patella and 54% the left. Patients who underwent CT scans for patella fractures were distributed across trauma-center levels as follows: the Level I trauma center treated 28 patients (2.8 patients/year); the Level II trauma center treated 155 patients (8.6 patients/year); and the Level III trauma centers treated 54 patients (3 patients/year), within the respective time-frames monitored.

### 3.2. AO/OTA Classification

The distribution of patella fractures according to the AO/OTA classification was as demonstrated in Table 1. Type A fractures accounted for 37 cases (15.6%), Type B fractures made up 71 cases (29.9%), and Type C fractures represented the majority with 129 cases (54.4%).

### 3.3. Significant Influence of the Level of Trauma Center on AO/OTA Classification

The AO/OTA classifications, spanning from A1 to C3, were numerically coded from 1 to 7, while trauma-center levels were assigned values from 1 to 3. In the linear regression model, the constant was 5.78, and the trauma-center level demonstrated a regression coefficient (β) of −1.32. This coefficient was associated with a t-value of −6.48 and a significance level of *p* < 0.001, indicating a statistically significant inverse relationship between trauma-center level and fracture classification.

### 3.4. Significant Influence of Age on AO/OTA Classification

The linear regression model furthermore demonstrated a significant relationship between age and the AO/OTA classification, with a regression coefficient (β) of 0.02, a t-value of 3.25 and a *p*-value of 0.001. This indicates that higher age is associated with a shift towards higher AO/OTA classification types.

### 3.5. No Influence of Sex on AO/OTA Classification

No statistically significant differences were observed in the distribution of fracture types between male and female patients. Furthermore, there was no significant association between sex and the level of trauma center.

### 3.6. Fracture Mapping

**Figure 5:**
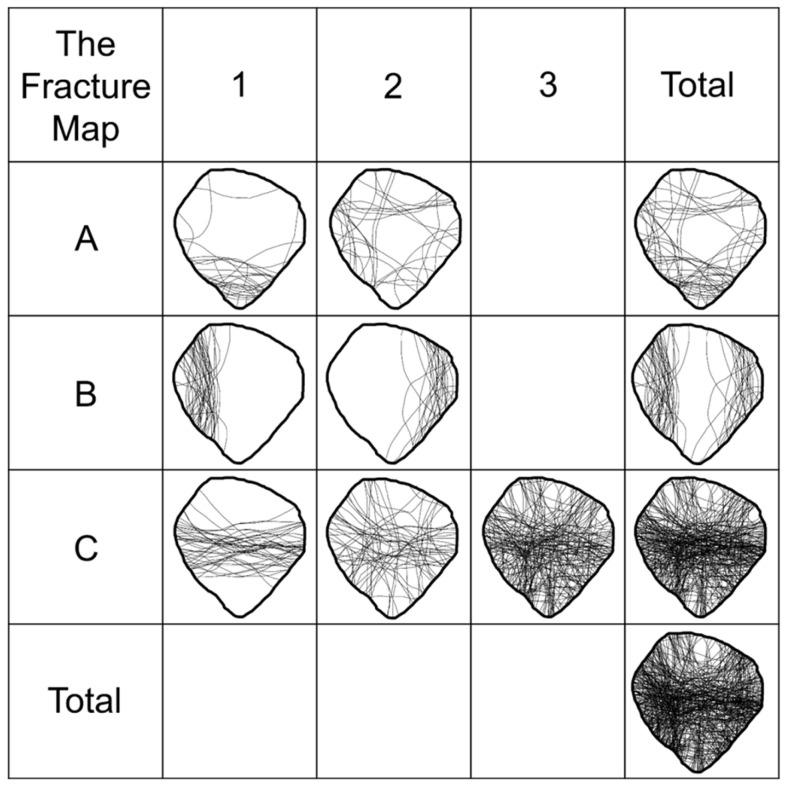
The fracture map.

The fracture mapping (see Figure 5) revealed consistent patterns across the cohort. The analysis demonstrated that fractures rarely involved the upper and lower poles of the patella. The highest density of fracture lines was observed in the central region, with a transverse orientation, extending medially and laterally.

## 4. Discussion

This study evaluated 237 patellar fractures using CT to identify patterns and influencing factors. The analysis revealed significant relationships between fracture severity (as per AO/OTA classification) and both the level of trauma center and patient age. While male patients were significantly younger than female patients, no differences in AO/OTA classification or trauma-center levels were observed between sexes. Fracture mapping demonstrated that the upper and lower poles of the patella were rarely affected, with the highest fracture density observed transversely in the central region.

### 4.1. Demographics

Male patients were significantly younger than female patients, with fractures nearly evenly distributed between the right and left patella. These findings align with existing literature demonstrating that fractures in older populations often involve females due to osteoporosis and age-related biomechanical changes [19,20,21]. The absence of laterality bias suggests no inherent biomechanical predisposition to injury on a particular side, consistent with general fracture pattern studies.

### 4.2. AO/OTA Classification

Type C fractures were the most prevalent, representing over half of all cases. This prevalence supports the established role of CT imaging in evaluating high-energy, complex fractures such as comminuted or multi-fragmentary injuries [19,21,22]. The distribution of fracture types also reflects the variability in injury mechanisms and the potential for more severe classifications in older, osteoporotic populations [23,24].

### 4.3. Significant Influence of the Level of Trauma Center on AO/OTA Classification

Trauma-center level significantly correlated with fracture severity, with less severe fractures more commonly treated at higher-level centers. This may be attributed to referral and triage patterns where more resource-intensive cases, such as C3 fractures, are directed to specialized centers for advanced surgical interventions [25,26]. These findings highlight the need for consistent triage protocols to optimize resource allocation.

### 4.4. Significant Influence of Age on AO/OTA Classification

Older patients presented with more severe fractures, as reflected in the linear regression results. This finding aligns with research indicating that reduced bone density and structural integrity in older adults predispose them to complex fractures following low-energy trauma [27,28]. The relationship between age and fracture severity reinforces the importance of tailoring treatment to older populations with diminished bone quality [29].

### 4.5. Fracture Mapping

Fractures predominantly affected the central patella in Type C fractures, sparing the upper and lower poles. This pattern aligns with biomechanical studies demonstrating that the central region of the patella bears the highest stress during direct or indirect trauma, making it the most common site of fracture propagation. In Type A and B fractures, the central region is less frequently involved, with a relative sparing of this area and a greater prevalence of fractures affecting the upper or lower poles. Using three-dimensional fracture mapping, Cho et al. identified the distal pole fragment as a relevant key fragment in more than 80% of cases and furthermore identified coronal split fragments in addition to distal pole fragments, or satellite fragments [30]. Interestingly, in the current study the highest density of fracture lines was observed in the central region of the patella. However, while Cho et al. only investigated AO/OTA type C fractures, type A and B fractures were deliberately included in the current study.

This distinction highlights the importance of considering fracture patterns when planning surgical interventions such as screw and/or locking plate osteosynthesis, cerclage wiring, or alternative methods [31,32,33,34]. The consistent mapping across patients supports the use of CT scan in localizing fracture sites for precise surgical planning [22,35,36].

### 4.6. Applications of Research

The findings of this study on patellar fractures have significant clinical and practical applications. Understanding the consistent patterns of fracture distribution and the demographic influences on fracture severity can guide improvements in diagnosis, treatment, and prevention strategies. The use of CT-based fracture mapping, as demonstrated, provides a valuable tool for personalized treatment planning. This method facilitates precise localization of fracture zones, aiding surgeons in selecting the most appropriate surgical approaches. For example, in Type C fractures with a high density of transverse fracture lines in the central patella, surgeons can prioritize techniques such as plate osteosynthesis to ensure robust fixation and minimize articular incongruity [37,38].

Additionally, the insights from fracture mapping have potential applications in developing biomechanical models that simulate patellar stress distribution during trauma. Such models could inform the design of improved protective gear for high-risk populations, such as athletes and laborers engaged in heavy physical activities [39]. While our findings provide insights into fracture distribution, the direct application to designing protective bracing remains speculative, as patellar fractures commonly result from both direct impact and indirect muscle-loading mechanisms. Furthermore, fracture mapping could be integrated into preoperative planning software, enabling virtual surgical simulations that improve accuracy and outcomes [40,41].

In the context of healthcare resource allocation, the correlation between trauma-center level and fracture severity underscores the importance of centralized care for complex cases. Policymakers and hospital administrators can leverage these findings to optimize triage protocols, ensuring patients with severe fractures are directed to specialized centers equipped for advanced surgical interventions. This approach can enhance patient outcomes while minimizing the burden on lower-level trauma centers.

Lastly, recognizing age-related trends in fracture severity underscores the need for targeted prevention and rehabilitation programs for older adults. Community-based initiatives aimed at fall prevention and osteoporosis management could play a crucial role in reducing both the incidence and severity of patellar fractures in this vulnerable population. In terms of rehabilitation, age-specific protocols that consider bone quality and healing capacity may enhance functional recovery and minimize long-term complications. While fracture mapping provides valuable insights into fracture patterns, its direct impact on rehabilitation protocols remains unclear. Given that rehabilitation outcomes are influenced by multiple factors, including surgical fixation stability and soft-tissue integrity, future prospective studies should investigate the relationship between fracture patterns and functional recovery.

### 4.7. Limitations

This study has some limitations. First, the decision to perform CT imaging was often based on fracture complexity, leading to an overrepresentation of complex fractures and potentially skewing the findings toward more severe injury patterns. Simpler fractures, often managed without CT, are likely underrepresented. A potential selection bias exists as CT scans were performed more frequently at Level I trauma centers due to polytrauma protocols, whereas at Level II and Level III centers, CT was likely reserved for complex fractures. This may have resulted in an overrepresentation of complex fractures in our dataset. Future prospective studies should aim to include a broader spectrum of fractures, including those diagnosed by radiographs alone [20].

Second, the inclusion of patients from a Level I trauma center, which primarily treats more severely injured individuals, introduces a selection bias that may not reflect the general population of patellar fractures. Existing literature suggests that polytrauma-related patellar fractures constitute a relatively small proportion (5–10%) of all patellar fractures. Given this, we believe our demographic analysis remains valid, as the majority of cases were isolated fractures rather than part of a polytrauma scenario. However, this aspect should be further validated in future studies [23].

Furthermore, our study primarily focused on coronal-plane CT fracture mapping. However, axial and sagittal imaging could provide further insights into non-displaced fracture lines and step-off deformities. Future studies should incorporate three-dimensional mapping techniques to comprehensively evaluate fracture morphology and displacement patterns as well as intraoperative results [22].

Moreover, inter-observer variability for AO/OTA classification was not formally assessed in this study. However, prior research indicates that CT-based classification significantly improves diagnostic accuracy and reliability compared to radiographs alone. Future studies should include inter-observer agreement analyses to validate classification reliability [10].

Additionally, while CT imaging provides detailed bony morphology, it does not assess cartilage damage and soft tissue injuries. MRI is crucial for evaluating associated ligamentous and cartilage lesions, which may influence treatment decisions. Future studies should integrate MRI to provide a more comprehensive assessment of patellar fracture injuries [12].

Lastly, as a retrospective study, variability in imaging protocols and clinical practices across centers may have affected the consistency of data. Future studies should aim for a more balanced inclusion of fracture types and prospective data collection to address these limitations.

## 5. Conclusions

This study demonstrates the central role of CT imaging in accurately classifying patellar fractures, with age and trauma-center level significantly influencing fracture severity. The consistent fracture mapping highlights the central region of the patella as the primary site of injury, sparing the upper and lower poles.

## Figures and Tables

**Table 1:** AO/OTA Classification.

AO/OTA Classification	1	2	3	Total
**A**	22 (9.3%)	15 (6.3%)		37 (15.6%)
**B**	42 (17.7%)	29 (12.2%)		71 (29.9%)
**C**	41 (17.3%)	36 (15.2%)	52 (21.9%)	129 (54.4%)
**Total**				237 (100%)

## Data Availability

Data are available from the corresponding author upon reasonable request.

## Abbreviations

The following abbreviations are used in this manuscript:

CT	Computed Tomography
ORIF	Open reduction internal fixation
AO	Arbeitsgemeinschaft für Osteosynthesefragen
OTA	Orthopaedic Trauma Association
